# Supplementary material for: The relationship between age and sex partner counts during the mpox outbreak in the UK, 2022
Source: PLoS One. 2023 Sep 8;18(9):e0291001. doi: 10.1371/journal.pone.0291001 (PMC10490899; doi:10.1371/journal.pone.0291001)
Supplement: S1 Appendix — (DOCX) [file pone.0291001.s001.docx]

## **S1.** Distribution of partner counts

Respondent counts (y-axis) for reported partner counts (x-axis). Columns are MSM, MSW or WSM. Rows are, within each partnership type, all population (top), age 45+ (middle), age 65+ bottom. All histograms are truncated to show only respondent counts when partners numbered > 1 (MSW, WSM, MSM age 65+) or > 5 partners (other MSMs)

MSM MSW WSM


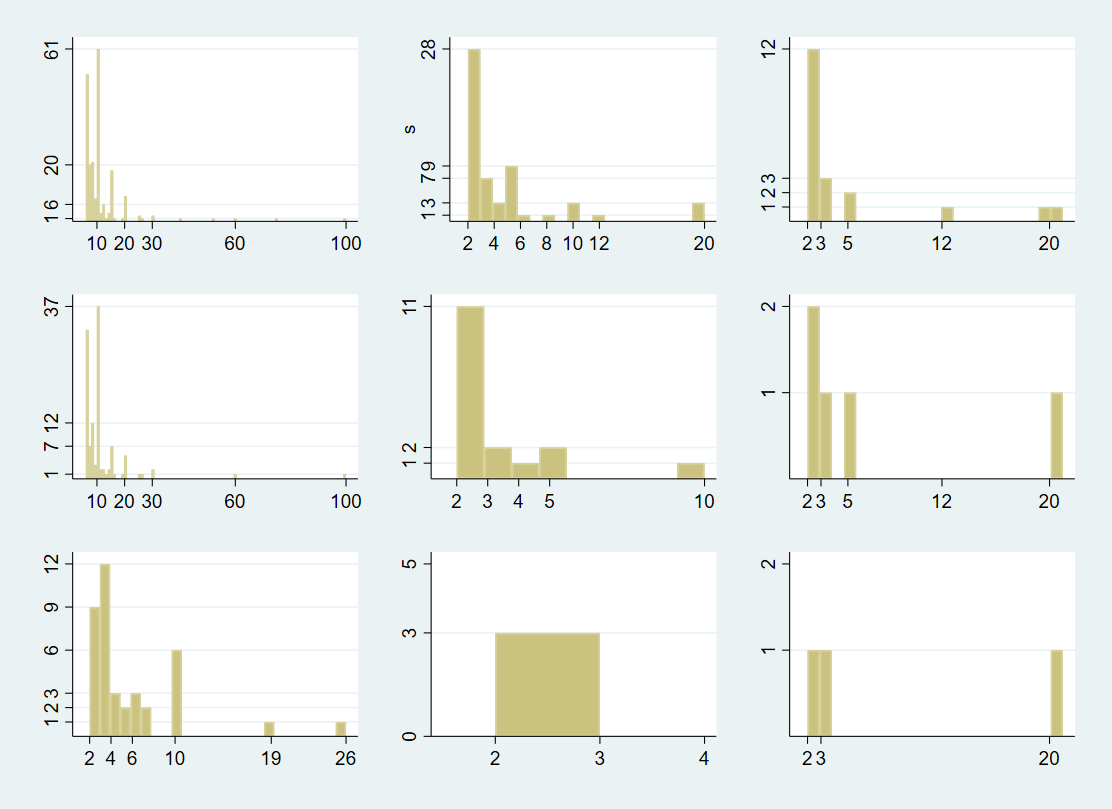


## All age

## Age 45+

## Age 65+

**Stata code to generate S1**

histogram D12A_1rec if candid_MSM == 1 & D12A_1rec > 5, bin(95) freq name(hist1a, replace) xtitle(“”) xlabel(10 20 30 60 100) ytitle("") ylabel(1 6 20 61)

histogram D12A_1rec if candid_MSM == 1 & D12A_1rec > 5 & Age >= 45, bin(95) freq name(hist1b, replace) xtitle(“”) xlabel(10 20 30 60 100) ytitle("") ylabel(1 7 12 37)

histogram D12A_1rec if candid_MSM == 1 & D12A_1rec > 1 & Age >= 65, bin(25) freq name(hist1c, replace) xtitle(“”) xlabel( 2 4 6 10 19 26) ytitle("") ylabel(1 2 3 6 9 12)

histogram D12B_1rec if candid_MSW == 1 & D12B_1rec > 1 , bin(19) freq name(hist2a, replace) xtitle("") xlabel( 2 4 6 8 10 12 20) ytitle("s") ylabel(1 3 7 9 28)

histogram D12B_1rec if candid_MSW == 1 & D12B_1rec > 1 & Age >= 45, bin(9) freq name(hist2b, replace) xtitle("") xlabel( 2 3 4 5 10) ytitle("") ylabel(1 2 11)

histogram D12B_1rec if candid_MSW == 1 & D12B_1rec > 1 & Age >= 65, bin(4) freq name(hist2c, replace) xtitle("") xlabel( 2 3 4) ytitle("") ylabel(0 3 5)

histogram D12A_1rec if candid_WSM == 1 & D12A_1rec > 1 , bin(21) freq name(hist3a, replace) xtitle("") xlabel( 2 3 5 12 20) ytitle("") ylabel(1 2 3 12)

histogram D12A_1rec if candid_WSM == 1 & D12A_1rec > 1 & Age >= 45 , bin(21) freq name(hist3b, replace) xtitle("") xlabel( 2 3 5 20) ytitle("") ylabel(1 2)

histogram D12A_1rec if candid_WSM == 1 & D12A_1rec > 1 & Age >= 65 , bin(21) freq name(hist3c, replace) xtitle("") xlabel( 2 3 20) ytitle("") ylabel(1 2)

graph combine hist1a hist2a hist3a hist1b hist2b hist3b hist1c hist2c hist3c, rows(3) cols(3)

## S2. Male partner counts of MSMs, previous 3 weeks, by sample

| S*ample:*  *#Partners* | Savanta General Population | Savanta UKMSM boost | Facebook & Instagram (Meta) | Grindr | Total |
| --- | --- | --- | --- | --- | --- |
| 0 | 44 | 92 | 273 | 205 | 614 |
| 1 | 92 | 85 | 295 | 167 | 639 |
| 2 | 15 | 14 | 119 | 126 | 274 |
| 3 | 3 | 4 | 83 | 83 | 173 |
| 4 |  | 3 | 46 | 60 | 109 |
| 5 | 3 | 1 | 58 | 47 | 109 |
| 6 | 2 | 3 | 23 | 24 | 52 |
| 7 |  |  | 13 | 7 | 20 |
| 8 | 1 | 1 | 10 | 9 | 21 |
| 9 |  |  | 4 | 4 | 8 |
| 10 | 1 | 2 | 27 | 31 | 61 |
| 11 |  |  | 2 | 1 | 3 |
| 12 |  | 2 | 3 | 1 | 6 |
| 13 |  |  | 1 |  | 1 |
| 14 |  | 1 | 2 |  | 3 |
| 15 |  |  | 11 | 7 | 18 |
| 16 |  |  | 1 |  | 1 |
| 19 |  |  |  | 1 | 1 |
| 20 |  |  | 4 | 5 | 9 |
| 25 |  |  | 2 |  | 2 |
| 26 |  |  |  | 1 | 1 |
| 30 |  |  | 1 | 1 | 1 |
| 40 |  |  | 1 |  | 1 |
| 52 |  |  |  | 1 | 1 |
| 60 |  |  |  | 1 | 1 |
| 75 |  |  |  | 1 | 1 |
| 100 |  |  | 1 |  |  |
| N= | 161 | 208 | 980 | 783 | 2132 |

## S3. Female partner counts of MSWs, previous 3 weeks, by sample

| *Sample*  #Partner | Savanta Gen Pop | Savanta UKMSM boost | Facebook & Instagram (Meta) | Grindr | Total |
| --- | --- | --- | --- | --- | --- |
| 0 | 396 | 14 | 50 | 81 | 541 |
| 1 | 548 | 7 | 20 | 29 | 604 |
| 2 | 16 | 3 | 4 | 5 | 28 |
| 3 | 3 | 2 |  | 2 | 7 |
| 4 | 2 |  |  | 1 | 3 |
| 5 | 6 |  | 1 | 2 | 9 |
| 6 |  |  |  | 1 | 1 |
| 8 | 1 |  |  |  | 1 |
| 10 | 1 | 1 |  | 1 | 3 |
| 12 | 1 |  |  |  | 1 |
| 20 | 3 |  |  |  | 3 |
|  |  |  |  |  |  |
| N= | 977 | 27 | 75 | 122 | 1201 |
|  |  |  |  |  |  |

## S4. Male partner counts of MSMs, previous 3 weeks, by respondent’s age group

| *Age:*  *#Partners* | 18-24 | 25-34 | 35-44 | 45-54 | 55-64 | 65+ | Total |
| --- | --- | --- | --- | --- | --- | --- | --- |
| 0 | 34 | 87 | 129 | 151 | 139 | 74 | 614 |
| 1 | 29 | 101 | 171 | 166 | 105 | 67 | 639 |
| 2 | 12 | 54 | 64 | 74 | 61 | 9 | 274 |
| 3 | 7 | 36 | 38 | 48 | 32 | 12 | 173 |
| 4 | 8 | 21 | 25 | 31 | 21 | 3 | 109 |
| 5 | 9 | 13 | 25 | 40 | 20 | 2 | 109 |
| 6 |  | 6 | 14 | 18 | 11 | 3 | 52 |
| 7 | 2 | 7 | 4 | 3 | 2 | 2 | 20 |
| 8 | 2 | 2 | 5 | 8 | 4 |  | 21 |
| 9 |  |  | 5 | 1 | 2 |  | 8 |
| 10 | 1 | 7 | 16 | 19 | 12 | 6 | 61 |
| 11 |  | 1 |  | 1 | 1 |  | 3 |
| 12 | 1 |  | 3 | 1 | 1 |  | 6 |
| 13 |  |  |  |  | 1 |  | 1 |
| 14 |  | 1 |  | 1 | 1 |  | 3 |
| 15 | 1 | 2 | 8 | 3 | 4 |  | 18 |
| 16 |  |  |  | 1 |  |  | 1 |
| 19 |  |  |  |  |  | 1 | 1 |
| 20 |  | 2 | 2 | 4 | 1 |  | 9 |
| 25 |  |  | 1 |  | 1 |  | 2 |
| 26 |  |  |  |  |  | 1 | 1 |
| 30 |  |  |  | 1 | 1 |  | 2 |
| 40 |  |  | 1 |  |  |  | 1 |
| 52 |  |  | 1 |  |  |  | 1 |
| 60 |  |  |  | 1 |  |  | 1 |
| 75 |  |  | 1 |  |  |  | 1 |
| 100 |  |  |  | 1 |  |  | 1 |
|  |  |  |  |  |  |  |  |
| N= | 106 | 340 | 513 | 573 | 420 | 180 | 2132 |

## S5. Female partner counts of MSWs, previous 3 weeks, by respondent’s age group

| *Age*  *Partners* | 18-24 | 25-34 | 35-44 | 45-54 | 55-64 | 65+ | Total |  |
| --- | --- | --- | --- | --- | --- | --- | --- | --- |
| 0 | 42 | 50 | 77 | 82 | 114 | 176 | 541 |  |
| 1 | 45 | 90 | 109 | 81 | 108 | 171 | 604 |  |
| 2 | 7 | 7 | 3 | 6 | 2 | 3 | 28 |  |
| 3 | 1 | 2 | 2 | 1 | 1 |  | 7 |  |
| 4 | 1 | 1 |  | 1 |  |  | 3 |  |
| 5 | 2 | 3 | 2 | 2 |  |  | 9 |  |
| 6 |  |  | 1 |  |  |  | 1 |  |
| 8 | 1 |  |  |  |  |  | 1 |  |
| 10 |  | 1 | 1 | 1 |  |  | 3 |  |
| 12 |  |  | 1 |  |  |  | 1 |  |
| 20 | 2 | 1 |  |  |  |  | 3 |  |
|  |  |  |  |  |  |  |  |  |
| N= | 101 | 155 | 196 | 174 | 225 | 350 | 1201 |  |

## S6. Male partner counts of WSMs, previous 3 weeks, by respondent’s age group

| *Age*  *Partners* | 18-24 | 25-34 | 35-44 | 45-54 | 55-64 | 65+ | Total |  |
| --- | --- | --- | --- | --- | --- | --- | --- | --- |
| 0 | 29 | 56 | 66 | 97 | 127 | 242 | 617 |  |
| 1 | 58 | 169 | 111 | 157 | 87 | 89 | 671 |  |
| 2 | 3 | 4 | 3 |  | 1 | 1 | 12 |  |
| 3 | 1 | 1 |  |  |  | 1 | 3 |  |
| 5 |  | 1 |  | 1 |  |  | 2 |  |
| 12 | 1 |  |  |  |  |  | 1 |  |
| 20 |  | 1 |  |  |  |  | 1 |  |
| 21 |  |  |  |  |  | 1 | 1 |  |
|  |  |  |  |  |  |  |  |  |
| N= | 92 | 232 | 180 | 255 | 215 | 334 | 1308 |  |

## S7. Male partner counts of MSMs, previous 3 weeks, by respondent’s occupational group

| *Occup:*  *#Partners* | HMA | Int | Sup | SkilMan | Unsk | Unemp | Stud | Ret | Total |  |
| --- | --- | --- | --- | --- | --- | --- | --- | --- | --- | --- |
| 0 | 87 | 188 | 119 | 24 | 41 | 44 | 17 | 90 | 610 |  |
| 1 | 133 | 219 | 136 | 30 | 20 | 18 | 20 | 62 | 638 |  |
| 2 | 56 | 113 | 57 | 17 | 7 | 5 | 7 | 11 | 273 |  |
| 3 | 37 | 52 | 36 | 5 | 8 | 5 | 7 | 21 | 171 |  |
| 4 | 27 | 43 | 20 | 3 | 5 | 2 | 4 | 4 | 108 |  |
| 5 | 29 | 38 | 17 | 7 | 7 | 3 | 5 | 3 | 109 |  |
| 6 | 12 | 24 | 8 | 2 | 3 |  |  | 3 | 52 |  |
| 7 | 4 | 8 | 5 | 1 |  |  |  | 2 | 20 |  |
| 8 | 2 | 8 | 3 |  |  | 1 | 3 | 4 | 21 |  |
| 9 |  | 5 | 3 |  |  |  |  |  | 8 |  |
| 10 | 13 | 27 | 9 |  | 4 | 1 | 1 | 6 | 61 |  |
| 11 | 1 |  |  | 1 |  |  |  | 1 | 3 |  |
| 12 |  | 5 |  |  | 1 |  |  |  | 6 |  |
| 13 |  |  |  |  |  | 1 |  |  | 1 |  |
| 14 | 1 | 1 | 1 |  |  |  |  |  | 3 |  |
| 15 | 3 | 4 | 6 | 1 | 1 |  | 2 | 1 | 18 |  |
| 16 | 1 |  |  |  |  |  |  |  | 1 |  |
| 19 |  | 1 |  |  |  |  |  |  | 1 |  |
| 20 | 1 | 5 | 1 |  | 1 | 1 |  |  | 9 |  |
| 25 | 2 |  |  |  |  |  |  |  | 2 |  |
| 26 | 1 |  |  |  |  |  |  |  | 1 |  |
| 30 |  |  |  | 1 |  |  |  | 1 | 2 |  |
| 40 |  | 1 |  |  |  |  |  |  | 1 |  |
| 52 |  | 1 |  |  |  |  |  |  | 1 |  |
| 60 |  |  | 1 |  |  |  |  |  | 1 |  |
| 75 | 1 |  |  |  |  |  |  |  | 1 |  |
| 100 | 1 |  |  |  |  |  |  |  | 1 |  |
|  |  |  |  |  |  |  |  |  |  |  |
| N= | 412 | 743 | 422 | 92 | 98 | 81 | 66 | 209 | 2123 |  |

Notes for Tables S7-S9:

Occupation Key (with exemplar jobs)

HMA: High managerial, administrative or professional, e.g. doctor, lawyer, medium / large company director

Int: Intermediate managerial, administrative or professional, e.g. teacher, manager, accountant

Sup: Supervisor, administrative or professional, e.g. police officer, nurse, secretary, self-employed

SkilMan: Skilled manual worker, e.g. mechanic, plumber, electrician, lorry driver, train driver

Unsk: Semi-skilled or unskilled manual worker, e.g. waiter, factory worker, receptionist, labourer

HW: Housewife *(women only, males not subgrouped due to very small counts, < 15)*

Unemp: Unemployed

Stud: Student

Ret: Retired on a state &/or private pension

## S8. Female partner counts of MSWs, previous 3 weeks, by respondent’s occupational group

| *Occup:*  *#Partners* | HMA | Int | Sup | SkilMan | Unsk | Unemp | Stud | Ret | Total |  |
| --- | --- | --- | --- | --- | --- | --- | --- | --- | --- | --- |
| 0 | 48 | 106 | 92 | 47 | 45 | 37 | 12 | 151 | 538 |  |
| 1 | 55 | 142 | 105 | 89 | 56 | 20 | 8 | 128 | 603 |  |
| 2 | 3 | 11 | 5 | 6 |  |  | 1 | 2 | 28 |  |
| 3 | 1 | 2 | 1 |  | 2 |  | 1 |  | 7 |  |
| 4 |  |  | 2 | 1 |  |  |  |  | 3 |  |
| 5 | 1 | 1 | 3 | 3 |  |  |  |  | 8 |  |
| 6 |  | 1 |  |  |  |  |  |  | 1 |  |
| 8 |  | 1 |  |  |  |  |  |  | 1 |  |
| 10 | 1 | 1 |  |  | 1 |  |  |  | 3 |  |
| 12 | 1 |  |  |  |  |  |  |  | 1 |  |
| 20 |  | 1 | 1 | 1 |  |  |  |  | 3 |  |
|  |  |  |  |  |  |  |  |  |  |  |
| N= | 110 | 266 | 209 | 147 | 104 | 57 | 22 | 281 | 1196 |  |

## S9. Male partner counts of WSMs, previous 3 weeks, by respondent’s occupational group

| *Occup:*  *#Partners* | HMA | Int | Sup | SkilMan | Unsk | HW | Unemp | Stud | Ret | Total |  |
| --- | --- | --- | --- | --- | --- | --- | --- | --- | --- | --- | --- |
| 0 | 16 | 87 | 132 | 57 | 73 | 16 | 50 | 5 | 181 | 617 |  |
| 1 | 38 | 161 | 159 | 101 | 80 | 28 | 27 | 9 | 68 | 671 |  |
| 2 |  | 4 | 1 | 3 | 2 |  | 1 |  | 1 | 12 |  |
| 3 |  |  |  |  | 1 |  |  | 1 | 1 | 3 |  |
| 5 |  | 1 |  |  |  |  |  | 1 |  | 2 |  |
| 12 |  | 1 |  |  |  |  |  |  |  | 1 |  |
| 20 | 1 |  |  |  |  |  |  |  |  | 1 |  |
| 21 |  |  |  |  |  |  |  |  | 1 | 1 |  |
|  |  |  |  |  |  |  |  |  |  |  |  |
| N= | 55 | 254 | 292 | 161 | 156 | 44 | 78 | 16 | 252 | 1308 |  |

## S10. Incidence risk ratios (95%CI) in univariate models, pooled recruitment samples

| Correlate \ Group | MSM | MSW | WSM |
| --- | --- | --- | --- |
| *Age (linear)* | 0.997 (0.99-1.00) | **0.98 (0.98-0.99)*** | **0.98 (0.97-0.98)*** |
|  |  |  |  |
| *Deprivation quintiles* | p = 0.027 | p = 0.34 | p = 0.37 |
| 1 – highest | 1.0 (ref) | 1.0 (ref) | 1.0 (ref) |
| 2 | 1.16 (0.99-1.37) | 1.12 (0.89-1.40) | 0.99 (0.80-1.21) |
| 3 | 0.95 (0.79-1.13) | **1.28 (1.02-1.61)** | 0.87 (0.69-1.09) |
| 4 | 1.00 (0.84-1.21) | 1.08 (0.84-1.37) | 1.08 (0.87-1.34) |
| 5 – lowest | 0.85 (0.69-1.04) | 1.10 (0.86-1.40) | 0.86 (0.67-1.10) |
|  |  |  |  |
| *BAME identity* | **1.23 (1.00-1.51)** | **2.04 (1.50-2.76)*** | 1.01 (0.71-1.43) |
|  |  |  |  |
| *Has university degree* | **1.16 (1.03-1.30)** | 1.12 (0.96-1.31) | **1.18 (1.01-1.38)** |
|  |  |  |  |
| *Has dependent child* | **0.75 (0.59-0.94)** | **1.63 (1.38-1.93)*** | **1.71 (1.48-1.98)*** |
|  |  |  |  |
| *Has regular partner* | 0.99 (0.88-1.04) | **1.64 (1.39-1.93)*** | **2.51 (2.11-2.99)*** |
|  |  |  |  |
| *Difficulty paying bills (very/extreme)* | **1.19 (1.00-1.41)** | **1.47 (1.23-1.75)*** | 0.97 (0.81-1.15) |
|  |  |  |  |
| *Region* | p < 0.001 | p < 0.001 | p < 0.001 |
| East Midlands | 0.99 (0.74-1.32) | 0.70 (0.49-1.01) | 0.94 (0.69-1.34) |
| East of England | **0.72 (0.52-0.91)*** | **0.70 (0.51-0.97)** | 1.13 (0.84-1.51) |
| London | 1.0 (ref) | 0.89 (0.67-1.18) | **2.03 (1.51-2.73)*** |
| North East | **0.42 (0.28-0.63)*** | 1.02 (0.70-1.49) | 1.01 (0.66-1.54) |
| North West | **0.65 (0.52-0.81)*** | 0.72 (0.53-0.98) | 1.10 (0.82-1.47) |
| Northern Ireland | 0.65 (0.37-1.12) | 0.84 (0.47-1.52) | 0.99 (0.51-1.94) |
| Scotland | **0.61 (0.47-0.78)*** | **0.65 (0.47-0.91)** | 0.92 (0.65-1.31) |
| South East | 0.81 (0.67-0.97) | 1.0 (ref) | 1.0 (ref) |
| South West | 0.76 (0.60-0.97) | **0.63 (0.45-0.88)*** | 0.79 (0.55-1.13) |
| Wales | 0.90 (0.65-1.23) | **0.54 (0.35-0.83)*** | 1.24 (0.87-1.76) |
| West Midlands | 0.89 (0.69-1.14) | 0.77 (0.55-1.07) | 1.21 (0.89-1.64) |
| Yorkshire & Humber | 0.93 (0.71-1.21) | 0.86 (.63-1.17) | 1.00 (0.72-1.39) |
|  |  |  |  |
| *Occupational group* | p < 0.001 | p < 0.001 | p < 0.001 |
| HMAP | 1.16 (0.99-1.35) | **1.76 (1.30-2.38)*** | 1.91 (1.39-2.62) |
| Int | 1.0 (ref) | **1.75 (1.38-2.23)*** | **1.33 (1.07-1.65)** |
| Sup | 0.87 (0.74-1.02) | **1.64 (1.27-2.12)*** | 1.0 (ref) |
| Skilled | 0.86 (0.65-1.14) | **2.03 (1.55-2.65)*** | 1.20 (0.94-1.55) |
| Semi/unskilled | 0.88 (0.66-1.15) | **1.47 (1.07-2.03)** | 1.01 (0.77-1.32) |
| Unemp | **0.57 (0.41-0.78)*** | 0.75 (0.45-1.23) | 0.67 (0.45-1.01) |
| Student | 0.95 (0.69-1.32) | 1.26 (0.67-2.36) | 1.93 (1.13-3.27) |
| Retired/KH | **0.67 (0.54-0.83)*** | 1.0 (ref) | 0.55 (0.57-0.95) |
|  |  |  |  |

Notes: Reference group was the largest for each partnership type (eg., London for MSMs and South East region for WSMs and MSWs). Age in years. BAME = Black, Asian or other minority ethnicity; *Occupation* *groups*: HMAP: Higher managerial or administrative professional, Int: Intermediate managerial or administrative professional; Sup: Supervisor, administrative or uniformed professional; Other: skilled, semi-skilled manual or unskilled workers; Unemp: unemployed; Student: university or college; Retired/KH: retired or keeping house. All models in this table were constructed using negative binomial regression, dependant variable in all models was category of partner counts (see Table 1). Significance denoted with **bold font**: p< 0.05 , ***:** p < 0.01.

***Relevant to model construction***

**Stata code** to calculate reciprocal age * population mean age for each partnership type, excluding zeros or not

drop MSMrecipAge nzMSMrecipAge

summarize Age if candid_MSM == 1

gen MSMrecipAge = `r(mean)' / Age if candid_MSM == 1

summarize Age if candid_MSM == 1 & D12A_1rec > 0

gen nzMSMrecipAge = `r(mean)' / Age if candid_MSM == 1 & D12A_1rec > 0

drop MSWrecipAge nzMSWrecipAge

summarize Age if candid_MSW == 1

gen MSWrecipAge = `r(mean)' / Age if candid_MSW == 1

summarize Age if candid_MSW == 1 & D12B_1rec > 0

gen nzMSWrecipAge = `r(mean)' / Age if candid_MSW == 1 & D12B_1rec > 0

drop WSMrecipAge nzWSMrecipAge

summarize Age if candid_WSM == 1

gen WSMrecipAge = `r(mean)' / Age if candid_WSM == 1

summarize Age if candid_WSM == 1 & D12A_1rec > 0

gen nzWSMrecipAge = `r(mean)' / Age if candid_WSM == 1 & D12A_1rec > 0

**Stata code** to generate survival series for Weibull regression

drop nzmaleptrs nzfemaleptrs

gen nzmaleptrs = D12A_1rec if D12A_1rec > 0

gen nzfemaleptrs = D12B_1rec if D12B_1rec > 0

**Stata code to generate BIC values in Table 2**:

nbreg D12A_1rec Age if candid_MSM == 1, irr

estat ic

nbreg D12A_1rec c.Age##c.Age if candid_MSM == 1, irr

testparm c.Age##c.Age

estat ic

nbreg D12A_1rec MSMrecipAge if candid_MSM == 1, irr

estat ic

nbreg D12A_1rec Age Education ib3.QRegion ib2.Occup_group ib1.IMD_quintile ib1.D1B ib1.Ethnicity_three Partnered DepChild if candid_MSM == 1, irr

estat ic

nbreg D12A_1rec c.Age##c.Age Education ib3.QRegion ib2.Occup_group ib1.IMD_quintile ib1.D1B ib1.Ethnicity_three Partnered DepChild if candid_MSM == 1, irr

estat ic

testparm c.Age##c.Age

nbreg D12A_1rec MSMrecipAge Education ib3.QRegion ib2.Occup_group ib1.IMD_quintile ib1.D1B ib1.Ethnicity_three Partnered DepChild if candid_MSM == 1, irr

estat ic

stset nzmaleptrs

streg Age if candid_MSM == 1, d(weibull)

estat ic

streg c.Age##c.Age if candid_MSM == 1, d(weibull)

estat ic

testparm c.Age##c.Age

streg nzMSMrecipAge if candid_MSM == 1, d(weibull)

estat ic

stset nzmaleptrs

streg Age Education ib3.QRegion ib2.Occup_group ib1.IMD_quintile ib1.D1B ib1.Ethnicity_three Partnered DepChild if candid_MSM == 1, d(weibull)

estat ic

streg c.Age##c.Age Education ib3.QRegion ib2.Occup_group ib1.IMD_quintile ib1.D1B ib1.Ethnicity_three Partnered DepChild if candid_MSM == 1, d(weibull)

estat ic

testparm Age

streg nzMSMrecipAge Education ib3.QRegion ib2.Occup_group ib1.IMD_quintile ib1.D1B ib1.Ethnicity_three Partnered DepChild if candid_MSM == 1, d(weibull)

estat ic

**Stata code to generate BIC values in Table 3**:

nbreg D12B_1rec Age if candid_MSW == 1, irr

estat ic

nbreg D12B_1rec c.Age##c.Age if candid_MSW == 1, irr

testparm c.Age##c.Age

estat ic

nbreg D12B_1rec MSWrecipAge if candid_MSW == 1, irr

estat ic

nbreg D12B_1rec Age Education ib3.QRegion ib2.Occup_group ib1.IMD_quintile ib1.D1B ib1.Ethnicity_three Partnered DepChild if candid_MSW == 1, irr

estat ic

nbreg D12B_1rec c.Age##c.Age Education ib3.QRegion ib2.Occup_group ib1.IMD_quintile ib1.D1B ib1.Ethnicity_three Partnered DepChild if candid_MSW == 1, irr

estat ic

testparm c.Age##c.Age

nbreg D12B_1rec MSWrecipAge Education ib3.QRegion ib2.Occup_group ib1.IMD_quintile ib1.D1B ib1.Ethnicity_three Partnered DepChild if candid_MSW == 1, irr

estat ic

stset nzfemaleptrs

streg Age if candid_MSW == 1, d(weibull)

estat ic

streg c.Age##c.Age if candid_MSW == 1, d(weibull)

estat ic

testparm c.Age##c.Age

streg nzMSWrecipAge if candid_MSW == 1, d(weibull)

estat ic

stset nzfemaleptrs

streg nzfemaleptrs Age Education ib3.QRegion ib2.Occup_group ib1.IMD_quintile ib1.D1B ib1.Ethnicity_three Partnered DepChild if candid_MSW == 1, d(weibull)

estat ic

streg nzfemaleptrs c.Age##c.Age Education ib3.QRegion ib2.Occup_group ib1.IMD_quintile ib1.D1B ib1.Ethnicity_three Partnered DepChild if candid_MSW == 1, d(weibull)

estat ic

testparm c.Age##c.Age

streg nzfemaleptrs nzMSWrecipAge Education ib3.QRegion ib2.Occup_group ib1.IMD_quintile ib1.D1B ib1.Ethnicity_three Partnered DepChild if candid_MSW == 1, d(weibull)

estat ic

**Stata code to generate BIC values in Table 4**:

nbreg D12A_1rec Age if candid_WSM == 1, irr

estat ic

nbreg D12A_1rec c.Age##c.Age if candid_WSM == 1, irr

testparm c.Age##c.Age

estat ic

nbreg D12A_1rec WSMrecipAge if candid_WSM == 1, irr

estat ic

nbreg D12A_1rec Age Education ib3.QRegion ib2.Occup_group ib1.IMD_quintile ib1.D1B ib1.Ethnicity_three Partnered DepChild if candid_WSM == 1, irr

estat ic

nbreg D12A_1rec c.Age##c.Age Education ib3.QRegion ib2.Occup_group ib1.IMD_quintile ib1.D1B ib1.Ethnicity_three Partnered DepChild if candid_WSM == 1, irr

estat ic

testparm c.Age##c.Age

nbreg D12A_1rec WSMrecipAge Education ib3.QRegion ib2.Occup_group ib1.IMD_quintile ib1.D1B ib1.Ethnicity_three Partnered DepChild if candid_WSM == 1, irr

estat ic

stset nzmaleptrs

streg Age if candid_WSM == 1, d(weibull)

estat ic

streg c.Age##c.Age if candid_WSM == 1, d(weibull)

estat ic

testparm c.Age##c.Age

streg nzWSMrecipAge if candid_WSM == 1, d(weibull)

estat ic

stset nzmaleptrs

streg Age Education ib3.QRegion ib2.Occup_group ib1.IMD_quintile ib1.D1B ib1.Ethnicity_three Partnered DepChild if candid_WSM == 1, d(weibull)

estat ic

streg c.Age##c.Age Education ib3.QRegion ib2.Occup_group ib1.IMD_quintile ib1.D1B ib1.Ethnicity_three Partnered DepChild if candid_WSM == 1, d(weibull)

estat ic

testparm Age

streg nzWSMrecipAge Education ib3.QRegion ib2.Occup_group ib1.IMD_quintile ib1.D1B ib1.Ethnicity_three Partnered DepChild if candid_WSM == 1, d(weibull)

estat ic
